# Magnitude and Associated Factors of Suicidal Ideation and Attempts among High School Adolescents of Jimma Town, Ethiopia

**DOI:** 10.4314/ejhs.v33i6.14

**Published:** 2023-11

**Authors:** Hayat Mohamed Aliy, Habtamu Abebe Getahun, Lelisa Sena Dadi

**Affiliations:** 1 School of Public Health, College of Medicine and Health sciences, Mizan-Tepi University, Mizan, Ethiopia; 2 Department of Epidemiology, Faculty of Public Health, Jimma University, Jimma, Ethiopia

**Keywords:** Suicidal ideation, Suicidal attempt, Adolescents, Jimma, Ethiopia

## Abstract

**Background:**

Studies show that suicidal ideation and attempt are major predictors of suicide. Flourishing technologies such cyber bullying, increased local and global events, like pandemics, wars, and effects of climate change exacerbate vulnerability of adolescents to mental health problems. Thus, timely epidemiological information is important for evidence-based practices. Therefore, the aim of this study was to assess the magnitude and associated factors of suicidal ideation and suicidal attempt among school adolescents.

**Methods:**

A school-based cross-sectional study was conducted in June 2022 on randomly selected 1144 school adolescents using multistage sampling technique. Data were collected using a self-administered questionnaire. Then, data were cleaned, entered into Epi-data V.3.1 and analyzed using SPSS version 26. Multivariable logistic regression was done to identify predictors of suicidal ideation and suicidal attempt among adolescents. Adjusted odds ratio and confidence interval (CI) were respectively used to measure statistical associations and their statistical significance.

**Results:**

The prevalence of lifetime suicidal ideation and attempt were 22.5%, and 13.3%, respectively, while 12-month suicidal ideation and attempt were found to be 14.6% and 10%, respectively. Being female, disappointment in school results, family history of suicide attempt, current alcohol intake, anxiety, and chronic medical condition were significantly associated with both suicidal ideation and attempt while cyber bullying was significantly associated with suicidal ideation only.

**Conclusions:**

Unsupportive home environment plus behavioral and medical conditions predispose school adolescents to suicidal ideation and attempt. The Ministry of Education and school administrations should facilitate favorable environment that enhance mental health awareness and protection of school adolescents. Building better parent-child relationship and parental discretion on the use of mobile phones can mitigate suicidal ideation and attempt.

## Introduction

Suicide is a major public health problem among adolescents, particularly among teenagers (1). Adolescence period is a developmental transition characterized by biological, cognitive, and psychosocial changes coinciding with high desire of experimenting new behaviors, low level of risk perception, and demanding independence and self-identity (2), which predispose the adolescents to high level risk of suicidal behaviors. Consequently, the level of mental problem among adolescents is noted to be high, ranging from 10% to 20%; yet, remains underdiagnosed and untreated due to several factors (2,3).

The World Health Organization (WHO) mental health action plan was devised to decrease the country-based suicide rate by 10% in 2020 (4). However, WHO estimates of 2019 showed the plan was not yet achievable given the slow progress of every country. Thus, the action plan was revised and extended to 2030 with increased target of decreasing suicide rates by one-third (5,6). Ethiopia has developed a mental health strategy in which suicide prevention has been considered as one of the key components. However, in the mental health atlas of WHO (7), the strategy was commented for lacking child and adolescent specific mental health strategy.

There are some studies regarding the magnitude of suicidal ideation and suicidal attempt among adolescents. A pooled analysis of study findings published from 2003-2017 revealed that the prevalence of suicidal ideation among school adolescents varies among countries (8)). In general, the trend of suicide ideation, and suicide attempt have been reported to have decreased or stabilized in some countries (9,10); on the other hand, increasing or fluctuating trends have also been reported (11,12). Still, suicidal ideation and anxiety were reported to be the highest in the African Region (13) while prevention efforts to alleviate the problem remains insignificant in the developing countries owing to numerous factors (1,9,13).

Low level socioeconomic status, female gender, lack of close friends and excessive parental control (5,6), lack of social support, living out of parents, disappointment with school results, history of bullying, sexual abuse, and alcohol intake, drugs abuse, and marijuana use are factors associated with suicidal ideation and attempt (11,12,14–16). Recently witnessed local and global environmental changes (17), coupled with drastically increased urbanization and globalization (18,19), have exacerbated the problem of mental disorders among adolescents. Excessive use of electronic media following flourishing technologies has further amplified the challenges of adolescent mental health (20). Furthermore, the pandemic of COVID-19 has also changed the decreasing trend of mental behaviors even in the developed countries such as USA (21) and Taiwan (22).

There have also been some studies (23–25) in Ethiopia regarding suicidal behaviors in adolescents. However, issues like cyber bullying were not addressed in those studies while studies elsewhere show that cyber victimization is associated with higher rates of suicidal behavior (3,20). The possible change in adolescent magnitude of suicidal ideation and attempt parallel to time change, technological advancements, and other globalization effects also imply the need for periodical evaluation and updated information to support policy and decision-makers. Therefore, the aim of this study was to assess the magnitude of suicidal ideation and suicidal attempt along with their associated factors among high school adolescents of Jimma Town.

## Methods

**Study setting and period**: The study was conducted in June 2022 in Jimma Town, which is found 357 kilometers to the southwest of Addis Ababa. The town has an estimated total population of 244,000 and 16 high-schools (eight are public and eight private) with student population of 16,189 (7,194 males and 8995 females).

**Study design**: A school-based cross-sectional design was employed to assess suicidal ideation and suicidal attempt among high school adolescents.

**Figure 1:**
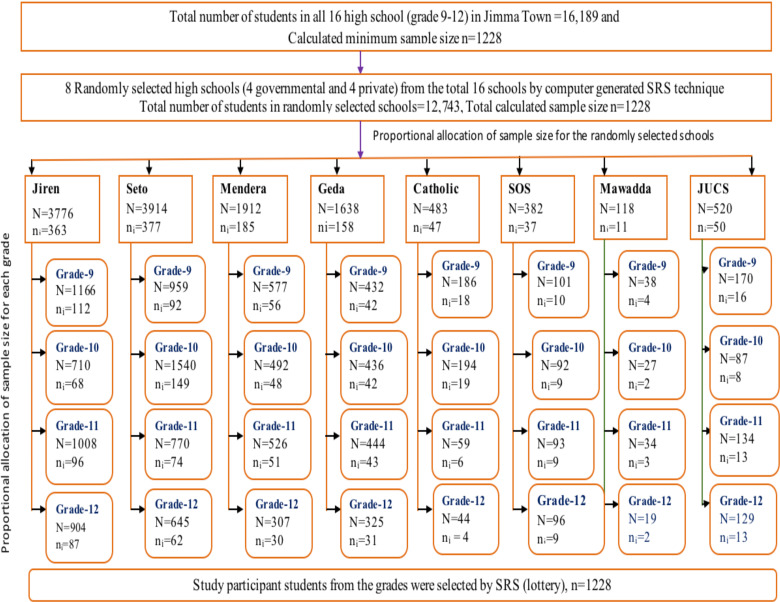
Schematic presentation of sampling procedure of study participants

**Sample size estimation and sampling procedure**: Sample size was estimated using Epi-info version 7 considering single population proportion formula, 95% confidence level, 3% margin of error, design effect of 1.5, non-response rate of 10%, and proportion of suicidal ideation (P = 0.225) (24). Therefore, the sample size was estimated to be 1228. Multistage sampling was applied to select the study participants. Initially, eight high schools (four governmental and four private) were selected by computer generated simple random sampling technique. Afterwards, sections of grades were also randomly identified. Then, the sample was proportionally allocated to the schools, grades, and sections. Finally, respondent adolescents were also randomly selected from attendance lists (Figure 1).

**Data collection tool and quality assurance**: Data was collected using a self-administered semi-structured questionnaire adopted from different literatures (8,24,25). Suicidal ideation and attempt were assessed using questions adopted from the WMH-CIDI questionnaire. Social support was assessed by OSLO social support scale-3 (OSSS-3) (26); depression and anxiety were assessed using DASS-21 scale. Most of these scales were validated in Ethiopia (27) and African settings (28,29). The modified sections of the questionnaire (anxiety and depression) were checked for consistency where Cronbach's alpha values were 0.767 and 0.791 for anxiety and depression, respectively. Wealth index was assessed using the EDHS-2016 wealth index questionnaire. The questionnaire was translated into Amharic and Afan-Oromo. Then, it was back-translated into English for checking consistency. Data was collected by six trained nurses who had experience in data collection. Additionally, two health officers supervised the data collection activities. The questionnaire was pre-tested in Agaro High School on 61 students, which was not included into the final analysis.

**The following operational definitions were used.**

**Chronic medical conditions**: It is a health condition or disease that is persistent or otherwise lasting (for more than three months) in its effect or disease that comes with time.

**Cyber bullying**: This term refers to bullying by email, text message, instant messaging, social networking, and other websites. Operationally, cyber bullying was defined if a participant student answered “yes” to the question whether s/he has ever experienced cyber bullying during the past 12 months.

**Statistical analysis**: The data was checked, entered into Epi-data version 3.1 and cleaned. Then, it was exported into SPSS version 26 for analysis. Descriptive statistics (frequencies, mean, and standard deviation) was used, and the prevalence of suicide ideation and suicide attempt were computed. Household relative wealth index (RWI) was analyzed using principal component analysis. The sum of resulting components was transformed into quantiles considering the EDHS classification of wealth index.

Factors that were found to be significantly associated with suicidal ideation and attempt at p-value ≤ 0.25 on bivariate analysis were entered into multivariable logistic regression to control possible confounders whereby imputation by mode was used for missing values. A p-value of ≤ 0.05 was considered to declare statistical significance. Adjusted odds ratio (AOR) along with its corresponding confidence interval (CI) was used to depict the strength of statistical association between predictor and outcome variables.

**Ethics approval and consent to participate**: Ethical clearance was obtained from Jimma University Institute of Health Institutional Review Board. Parents/guardians' informed consents were obtained for students under the age of 18 years where the consent forms were sent to the parents/guardians via the students for signature, and assents of the children were also obtained to participate. Informed written consents of students of 18 years or above were also secured before the commencement of data collection. Study participants were kept to sit far apart to keep confidentiality. The respondents were also instructed not to write their names on the questionnaire to maintain anonymity. Contact numbers of a licensed psychiatrist and advices were given to all study participants so that only those who responded “Yes” to suicidal behavior questions (question numbers were mentioned) should consult the psychiatrist.

## Results

**Socio-demographic characteristics of the study population**: A total of 1144 students participated in the study (response rate 93.2%), and female participants were 713 (62.3%). The mean age of the respondents was about 17 years (16.9 ±1.96) while their age range was 15 to 19 years. The number of respondents from grades 9, 10, 11, and 12 constituted 310(27.1%), 347 (30.3%), 293 (25.6%) and 194 (17.0%), respectively. More than two-third (71.2%) of the study participants lived with their parents while one-in-five (20.6%) lived with their relatives. Close to half (48.2%) and one-third (31.6%) of them were Muslims and Orthodox Christians, respectively. Regarding household relative wealth index, those in the 5^th^ and 4^th^ categories constituted only 20.2% and 11.5%, respectively (Table 1).

**Table 1:** Socio-demographic characteristics of study participants, Jimma Town, Southwest Ethiopia 2022 (n=1144)

Variable and Category	N (%)
Sex	
Male	431 (37.7)
Female	713 (62.3)
Age in years	
≤ 17 years	719 (62.8)
≥ 18 years	425 (37.2)
Grade	
9	310 (27.1)
10	367 (32.1)
11	293 (25.6)
12	194 (15.2)
Live with	
Parents	814 (71.2)
Relatives	236 (20.6)
Alone	57 (5.0)
Others*	37 (3.2)
Religion	
Orthodox	361 (31.6)
Muslim	551 (48.2)
Protestant	195 (17.0)
Others	37 (3.2)
Household Relative Wealth index	
Lowest	229 (20.0)
Low	286 (25.0)
Middle	267 (23.3)
High	131 (11.5)
Highest	231 (20.2)
Total	1144 (100)

* Others Spouse = 24 (2.1%) and non-relatives = 13 (3.2%)

**Psycho-social characteristics of respondents**: More than two-third (67.5%) of the study participants reported that they had been disappointed with their school results. Moreover, half (50.7%) of the participants reported difficulties of focusing while doing home works. Two-third (67.7%) of the participants perceived living in supportive family environments. However, only 44.2% of the respondents reported that the school environment was supportive, while 169(14.8%) reported experiencing bullying in the school environs. Cyber bullying was reported by 177 (15.5%) of the respondents, mainly by female students (9.44%). However, most (88.9%) of the students reported that they were not involved in physical violence behaviors. Some of the students (13.2%) had family history of suicidal attempt and loss of a family member (5.5%) due to suicide. Social support was perceived poor by 503(44.2%) of the adolescents (Table 2).

**Table 2:** School and Family Environment Related Experiences of study participants, Jimma Town, Southwest Ethiopia

Variable and Category	N (%)
**Disappointed with school results**	
No	372 (32.5)
Yes	772 (67.5)
**Had difficulties in focusing**	
No	564 (49.3)
Yes	580 (50.7)
**Experienced school bullying**	
No	975 (85.2)
Yes	169 (14.8)
**Involved in physical violence activities**	
No	1017 (88.9)
Yes	127 (11.1)
**School Environment**	
More supportive	506 (44.2)
Less supportive	638 (55.8)
**Family Environment**	
Non-supportive	37 (32.3)
Supportive	774 (67.7)
**Understanding of parents**	
No	333 (29.1)
Yes	811(70.9)
**Social support**	
Poor	503 (44)
Moderate	435 (38)
Strong	206 (18)
**Family history of suicide**	
No	1081 (94.5)
Yes	63 (5.5)
**Family history of suicidal attempt**	
No	993 (86.8)
Yes	151(13.2)
**Total**	**1144 (100)**

**Substance use and health-related experiences of the study participants**: Regarding substance use, 95(8.3%) and 53(4.6%) of the study participants reported history of smoking and current smoking, respectively. Similarly, 186(16.3%) and 97(8.5%) of the study participants reported lifetime and current alcohol consumption, respectively. Sexual abuse was reported by 75(6.6%) respondents (4.3% females and 2.2% males). Based on the DASS scale, only 662(57.9%) and 674(58.9%) of the respondents had normal scores of anxiety and depression, respectively Again, 174(15.2%) of the participants (11.1% females and 4.1% males) reported having a chronic medical condition. Lifetime suicidal ideation and history of suicidal attempts were reported by 267(23.3%) and 151(13.3%) of the respondents, respectively. The prevalence of suicidal ideation and suicidal attempts among the study participants within 12 months preceding the study were 167(14.6%) and 114(10.0) %, respectively (Table 3).

**Table 3:** Health related characteristics of study participants, Jimma town, South west Ethiopia 2022 (n=1144)

Variable and Category	Frequency (%)
**Ever/life time smoking**	
No	1049 (91.7)
Yes	95(8.3)
**Current smoking**	
No	1091 (95.4)
Yes	53 (4.6)
**Ever/life time alcohol use**	
No	958 (83.7)
Yes	186 (16.3)
**Currently alcohol use**	
No	1047 (91.5)
Yes	97 (8.5)
**Sexual abuse**	
No	1069 (93.4)
Yes	75 (6.6)
**Chronic medical condition**	
No	970 (84.8)
Yes	174 (15.2)
**Anxiety**	
Normal	662 (57.9)
Mild	91 (8.0)
Moderate	178 (15.5)
Severe	87 (7.6)
Extremely severe	126 (11.0)
**Depression**	
Normal	674 (58.9)
Mild	141 (12.3)
Moderate	202 (17.7)
Severe	64 (5.6)
Extremely severe	63 (5.5)
**Life time suicide ideation**	
No	877 (76.7)
Yes	267 (23.3)
**Suicide ideation in the last 12**	
**months**	
No	977 (85.4)
Yes	167 (14.6)
**Life time suicide attempt**	
No	993 (86.8)
Yes	151 (13.2)
**Suicide attempts in the last 12 months**	
No	1030 (90.0)
Yes	114 (10.0)

**Factors associated with suicidal ideation**: Factors such as participants' grades, household relative wealth index, persons lived with, facing hard time, being bullied by someone, social support, family history of suicide, smoking, sexual abuse, and religion were not significantly associated with suicidal ideation of the adolescents. On the other hand, gender, difficulty of focusing, cyber bullying, family environment, parent understanding, family history of suicidal attempt, alcohol drinking, anxiety (severe and extremely severe forms), and chronic medical conditions were significantly associated with suicidal ideation of the adolescents.

Female adolescents were about two times (AOR: 1.7; 95% CI: 1.1, 2.6) more likely to experience suicidal ideation compared to male adolescents. Similarly, adolescents who had difficulty of focusing (AOR: 1.6; 95% CI: 1.1, 2.5) and those who had non-supportive family environment (AOR: 1.7; 95% CI: 1.1, 1.6) were closely two times more likely to encounter suicidal ideation. Adolescents who were disappointed with school performance (AOR: 2.3; 95% CI: 1.4, 4.0) and those who faced cyber bullying (AOR: 2.2; 95% CI: 1.4, 3.4) were more than two times more likely to experience suicidal ideation compared to their counterparts. Adolescents who had family history of suicidal attempts were more than two times (AOR: 2.7; 95% CI: 1.7, 4.2) more likely to face suicidal ideation. Alcohol drinkers were more than three times (AOR: 3.6; 95% CI: 2.0, 6.4) more likely to encounter suicidal ideation compared to non-drinker.

School adolescents who had chronic medical conditions (AOR: 2.1; 95% CI: 1.3, 3.2) and those who did not have supportive family environment (AOR: 1.7; 95% CI: 1.1, 2.6) were about two times more likely to ideate suicide while those who had parental understanding were less (AOR: 0.6; 95% CI: 0.4, 0.9) likely to experience suicidal ideation. Those school adolescents who had severe and extremely severe anxiety were more than two (AOR: 2.4; 95% CI: 1.2, 5.0), and four (AOR: 4.1; 95% CI: 2.0, 8.3) times likely to experience suicidal ideation, respectively (Table 4).

**Table 4:** Factors associated with suicidal ideation among High School Adolescents, Jimma Town, Southwest Ethiopia (n=1144)

Variable	Category	Suicidal ideation		P-value	COR	AOR (95% CI)

		yes	No			
Sex	Male	46	385		1	
	Female	121	592	0.02	1.7	1.7 (1.1, 2.6)
Difficulties of	No	51	513		1	
focusing	Yes	116	464	0.029	2.5	1.6 (1.1, 2.5)
Disappointed in	No	23	349		1	
school results	Yes	144	628	0.002	3.5	2.3 (1.4, 4.0)
Cyber bullying	No	109	858		1	
	Yes	58	119	0.001	3.8	2.2 (1.4, 3.4)
Family history of	No	106	887		1	
suicidal attempt	Yes	61	90	0.000	5.7	2.7 (1.7, 4.2)
Current alcohol	No	128	919		1	
use	Yes	39	58	0.000	4.8	3.6 (2, 6.4)
Chronic medical	No	108	862		1	
condition	Yes	59	115	0.002	4.1	2.1 (1.3, 3.2)
Family	Non-supportive	91	279	0.020	3.0	1.7 (1.1, 2.6)
Environment	Supportive	76	698		1	
Understanding	No	88	245		1	
parents	Yes	79	732	0.015	0.3	0.6 (0.4, 0.9)
Anxiety	Normal	43	619		1	
	Mild	14	77	0.296	2.6	1.5 (0.7, 3)
	Moderate	30	148	0.237	2.9	1.5 (0.8, 2.7)
	Severe	24	63	0.019	5.5	2.4 (1.2-5)
	Extremely severe	56	70	0.000	11.5	4.1 (2, 8.3)
Depression	Normal	46	628		1	1
	Mild	27	114	0.083	3.2	1.7 (0.9, 3.3)
	Moderate	47	155	0.353	4.1	1.3 (0.7, 2.4)
	Severe	18	46	0.455	5.3	0.7 (0.3, 1.7)
	Extremely severe	29	34	0.050	11.6	2.3 (1.0, 5.2)

**Factors associated with suicidal attempts**: In the multivariable model, variables such as participants' grades, persons lived with, household relative wealth index, facing hard time, being bullied by someone, social support, cyber bullying, school support, understanding of parents, family history of suicide ideation, history of smoking, sexual abuse, and religion were not significantly associated with suicidal attempt of the adolescents. On the other hand, gender, difficulty of focusing, disappointment with school results, family environment, family history of suicidal attempt, alcohol drinking, anxiety (except the mild form), and chronic medical conditions were significantly associated with suicidal attempt of the adolescents.

Female school adolescents were two times (AOR: 2; 95% CI: 1.2, 3.4) more likely to attempt suicide compared to male adolescents. School adolescents who were disappointed by their school performance were two times (AOR: 3.2; 95% CI: 1.6, 6.5) more likely to attempt suicide. Those adolescents whose family environment was non-supportive were about two times (AOR: 1.8; 95%CI: 1.1, 2.9) more likely to attempt suicide. School adolescents who had family history of suicide were about three times (AOR: 2.9; 95%CI: 1.8, 4.9) more likely to attempt suicide while those who drank alcohol were closely five times (AOR: 4.8; 95% CI: 2.6, 8.8) more likely to attempt suicide compared to non-drinkers. Adolescents who had mild and moderate anxiety were about two times more likely to attempt suicide. Moreover, those who had severe and extremely severe anxiety were about three (AOR: 2.9; 95% CI: 1.4, 6.1) and more than five times (AOR: 5.5; 95% CI: 2.9, 10.3) more likely to attempt suicide, respectively, compared to normal adolescents. Adolescents who had chronic medical conditions were two times (AOR: 2.0; 95% CI: 1.2, 3.2) more likely to attempt suicide (Table 5).

**Table 5:** Factors associated with suicidal attempt among High School Adolescents, Jimma Town, Southwest Ethiopia (n =1144)

Variables	Categories	Suicidal attempt				AOR (95% CI)

		Yes	No	COR	P-value	
Sex	Male	28	403			1
	Female	86	627	2	0.010	2 (1.2, 3.4)
Disappointed in school	No	12	360	1		1
results	Yes	102	670	4.6	0.000	3.5 (1.8, 6.8)
Cyber bullying	No	75	892	1		1
	Yes	39	138	3.4	0.080	1.6 (0.9, 2.7)
Family Environment	Non-supportive	65	305	3.2	0.027	1.8 (1.1, 2.9)
	Supportive	49	725	1		1
Understanding parents	No	62	271	1		1
	Yes	52	759	0.3	0.057	0.6 (0.4, 1.0)
Family history of	No	67	926	1		1
suicidal attempt	Yes	47	104	6.2	0.000	2.9 (1.8, 4.9)
Current alcohol use	No	81	966	1		1
	Yes	33	64	6.1	0.000	4.8 (2.6. 8.8)
Anxiety	Normal	24	638	1		1
	Mild	10	81	3.3	0.060	2.2 (1.0, 5.0)
	Moderate	22	156	3.7	0.021	2.2 (1.1, 4.2)
	Severe	16	71	6	0.006	2.9 (1.4-6.1)
	Extremely severe	16	71	6	0.000	5.5 (2.9, 10.3)
Chronic medical	No	71	899	1		1
condition	Yes	43	131	4.2	0.008	2 (1.2, 3.2)

## Discussion

In this study, the lifetime prevalence of suicidal ideation was high, which is slightly higher than the findings of school-based studies done in central (25) and western (24) parts of Ethiopia. On the contrary, it was lower than the findings of a study in Poland (30), which can be due to socioeconomic, cultural, and sample size differences of the two studies. Regarding one-year prevalence of suicidal ideation, it is similar to the finding of Vietnam (31); however, it is lower than the findings of studies conducted in Mozambique (32), Swaziland (11), and USA (33) on large sample sizes. The difference can also be attributed to socioeconomic and cultural differences among the study populations.

According to the findings of this study, female students had experienced more suicidal ideation and suicidal attempt compared to male students. These results are consistent with studies done in different countries, including Ethiopia (25), Nigeria (34), Tunisia (35) (for suicidal ideation only), Swaziland (11), and a polled study finding of 90 countries (8). Such consistent findings can be reflective of gender difference where female adolescents encounter more life difficulties and are more prone to suicidal behaviors irrespective of socioeconomic level of countries.

Disappointment with exam results was associated with higher risk of suicidal ideation and attempt. Similarly, inability of staying focused while doing home works was also associated with higher odds of suicidal ideation. These findings are consistent with previous findings of studies done in in west and central Ethiopia (24,25) and Nigeria (12). Both the above factors can be attributed to the fact that failure to meet parental and societal expectations of good results can make adolescents view as their life is worthless and consider suicide as an option. Cyber bullying was also significantly associated with higher odds of suicidal ideation. This finding is consistent with findings in the studies done in Canada (36) and USA (3), which showed the global feature of cyber bullying. Cyber bullying can have detrimental effects on self-esteem of adolescents since the victims even might not know who attack them, and the anonymity makes it difficult to defend themselves from the attackers, which creates a sense of losing control.

Having parental understanding showed a significant protective association against suicidal ideation, while lack of supportive family environment was associated with increased risk of both suicidal ideation and suicidal attempts. These results are in line with a pooled study findings of 90 countries (8) and a finding from Utah, USA (37). This implies the importance of adolescents' belief in their parents understanding, which gives more chance of discussing possible worries and problems with their parents to seek solutions.

Family history of suicidal attempt is associated with increased risk of both suicidal ideation and suicidal attempt. Consistent findings were reported from Ethiopia (25) and Tunisia (35), implying the familial linkages of the behaviors. Current alcohol intake was significantly associated with increased risk of both suicide ideation and attempting it. However, this finding is contrary to the finding of western Ethiopia (24) possibly, due to the cultural commonness of alcohol use in the latter study's setting. On the other hand, consistent results were seen in a study done in South Korea (38) where alcohol use was significantly associated with a higher risk for suicidal ideation.

Adolescents who categorized in severe and extremely severe anxiety scores, and those who reported chronic medical condition were at higher risk of experiencing suicidal ideation and suicidal. These results are in line with study findings of other African countries (12,32,39,40). Such consistent findings in different settings might be due to the fact that chronic pain and restlessness can lead adolescents to hopeless and prone to suicidal behaviors as a means of pain-relief.

In conclusion, the prevalence of suicidal ideation and attempt were generally high among the school adolescents. It is consistent with other findings, which still needs due considerations since ideation and attempt are concepts directly related to mortality. In this regard, more vulnerable adolescents, that is, those who have history of family suicide attempt, cyber bullying, alcohol use, and anxiety need to be considered for future efforts to mitigate the problem. Therefore, strengthening school health (including mental health), building better relationship between adolescents and their parents/guardians, family-discretion, and continuous monitoring about the social media use of adolescents and responsible adolescent use of mobile phones are recommended to alleviate the thoughts of suicidal behaviors and their consequences.
